# Ultrafast strong-field dissociation of vinyl bromide: An attosecond transient absorption spectroscopy and non-adiabatic molecular dynamics study

**DOI:** 10.1063/4.0000102

**Published:** 2021-06-15

**Authors:** Florian Rott, Maurizio Reduzzi, Thomas Schnappinger, Yuki Kobayashi, Kristina F. Chang, Henry Timmers, Daniel M. Neumark, Regina de Vivie-Riedle, Stephen R. Leone

**Affiliations:** 1 Department of Chemistry, LMU Munich, 81377 Munich, Germany; 2 Department of Chemistry, University of California, Berkeley, California 94720, USA; 3 Chemical Sciences Division, Lawrence Berkeley National Laboratory, Berkeley, California 94720, USA; 4 Department of Physics, University of California, Berkeley, California 94720, USA

## Abstract

Attosecond extreme ultraviolet (XUV) and soft x-ray sources provide powerful new tools for studying ultrafast molecular dynamics with atomic, state, and charge specificity. In this report, we employ attosecond transient absorption spectroscopy (ATAS) to follow strong-field-initiated dynamics in vinyl bromide. Probing the Br M edge allows one to assess the competing processes in neutral and ionized molecular species. Using *ab initio* non-adiabatic molecular dynamics, we simulate the neutral and cationic dynamics resulting from the interaction of the molecule with the strong field. Based on the dynamics results, the corresponding time-dependent XUV transient absorption spectra are calculated by applying high-level multi-reference methods. The state-resolved analysis obtained through the simulated dynamics and related spectral contributions enables a detailed and quantitative comparison with the experimental data. The main outcome of the interaction with the strong field is unambiguously the population of the first three cationic states, *D*_1_, *D*_2_, and *D*_3_. The first two show exclusively vibrational dynamics while the *D*_3_ state is characterized by an ultrafast dissociation of the molecule via C–Br bond rupture within 100 fs in 50% of the analyzed trajectories. The combination of the three simulated ionic transient absorption spectra is in excellent agreement with the experimental results. This work establishes ATAS in combination with high-level multi-reference simulations as a spectroscopic technique capable of resolving coupled non-adiabatic electronic-nuclear dynamics in photoexcited molecules with sub-femtosecond resolution.

## I. INTRODUCTION

Since the demonstration of attosecond pulses, in the extreme ultraviolet (XUV) region of the electromagnetic spectrum (10–124 eV), via high-order harmonic generation (HHG), these pulses have been exploited for time-resolved investigations of ultrafast photoinitiated processes in atoms, molecules, and solids. Due to the high associated photon energy, capable of easily removing a valence electron from the sample under consideration, attosecond pulses allow one to create electron wave packets extremely well localized in time. This property, which makes them very sharp photo-triggering tools in a “pump-probe” scenario, is at the basis of the early attosecond streaking technique.^1^ Originally devised as a temporal characterization methodology for the attosecond pulses themselves,^2^ “streaking” has been exploited as a genuine spectroscopic tool, allowing for the determination of attosecond delays in photoemission both in atomic^3,4^ and solid-state^5,6^ systems. With the same spirit, attosecond pulses have been used in the last decade to create electron wave packets in highly excited cationic states of molecules, leading to the discovery of effects such as electron localization in diatomic molecules^7^ and, later, of purely electronic charge migration in biomolecules.^8^ On the other hand, the broad bandwidth of attosecond pulses makes them particularly valuable probing tools, because of the element, charge, and electronic state sensitivity gained by accessing the inner valence (in the XUV) and the core level states (in the soft x-ray region, and, possibly in the future, in the hard x ray) of elements. Along these lines, after the seminal work of Goulielmakis *et al.*,^9^ attosecond XUV pulses have been exploited to take snapshots of ultrafast processes in atoms,^10,11^ molecules,^12,13^ and solid-state materials,^14–17^ triggered by the strong-field interaction of the sample with an ultrashort few-cycle pulse. While this scheme is very convenient from the implementation viewpoint [carrier-envelope phase (CEP) stable few-cycle pulses are ideal drivers for the generation of isolated attosecond pulses, and thus, a replica for excitation purposes can be easily derived] and grants exquisite temporal localization of the initially prepared excited wave packet due to the extremely short pulses employed, there are two main bottlenecks historically ascribed to this methodology, limiting the range of applications—on the one hand, the non-resonant and non-perturbative nature of the excitation process; on the other, the complications in data interpretation due to multiplet effects in probing inner valence states (usually M edges) rather than genuine core level states (K and L edges). Some of the main recent directions in attoscience and, in general, of table-top ultrafast soft x-ray spectroscopy solve these methodological issues, moving toward single photon excitation schemes (from strong field to weak field)^18,19^ and core level probing (from 800-nm-driven XUV HHG to soft x-ray ponderomotively scaled HHG).^20–22^ Because of the great spectroscopic interest in the water window spectral region (containing the K absorption edges of carbon, nitrogen and oxygen as well as the L edges of calcium, scandium, titanium, and vanadium) for chemistry, biology, and materials science, these developments are crucial for the future of the field and hold promise to elucidate photoinduced processes of paramount importance, such as UV radiation damage of DNA and light-induced phase transitions from each individual atom's perspective. Still, the above-mentioned difficulties can be greatly diminished by an appropriate theoretical ansatz.

For the complete treatment of such time-resolved spectroscopy experiments in a theoretical framework, three different problem sets need to be addressed. As a first step, one has to describe the interaction of the system with the strong near infrared (NIR) field. Although it is possible^23–25^ to simulate the strong-field ionizations and excitations, it is not the focus of this work and we assume an instantaneous ionization or excitation. Second, one has to adequately describe the ultrafast processes the system undergoes after irradiation with the strong few-femtosecond NIR pump pulse. In our work, this is done by performing *ab initio* non-adiabatic molecular dynamics (NAMD) for the relevant electronic states. This makes it possible to resolve both the changes in the electronic structure and the nuclear motion over time. The third problem set is the simulation of the time-resolved XUV absorption spectrogram. In order to tackle this task, we use the restricted active space self-consistent field (RASSCF) theory followed by a perturbative inclusion of the dynamic electron correlation (RASPT2) to calculate the XUV absorption along each trajectory.

Besides the high-quality description of the absorption itself, the RASPT2 technique also allows one to decompose the entire spectrogram into its state-specific components. The RASPT2 ansatz to calculate the XAS spectra was first proposed by Josefsson and co-workers^26^ to calculate the L-edge spectra of transition metal complexes. Due to the versatility of the RASSCF, it was used by many groups in varying cases,^27–42^ but, in general, there are a variety of methods reported in the literature to calculate x-ray absorption (XAS) or XUV absorption spectra. First, simulations were done with the static exchange (STEX) approximation.^43–46^ Later, Stener and co-workers^47^ proposed an ansatz based on the restricted excitation window time dependent density functional theory (REW-TDDFT), which was successfully utilized in a number of different cases.^20,48–51^ More recently, the core-valence separation (CVS) approximation^52^ was used in conjunction with coupled-cluster (CC) theory.^53–57^ Also, Hait and co-workers successfully utilized the Restricted Open-Shell Kohn–Sham (ROXS) theory^58,59^ in the prediction of core-level spectra.^60,61^ A good overview of the various methods is given by Zhang and co-workers^62^ and, more recently, in the reviews by Norman and Dreuw^63^ and Lundberg and Delcey.^64^

Here, we propose a joint experimental and theoretical approach and apply it to follow the strong-field-initiated dynamics of vinyl bromide ( $C_{2}H_{3}\text{Br}$), exposed to a few-cycle NIR field. The ultrafast photophysics of vinyl bromide, similar to other substituted ethylenes, has been historically studied in connection to photoactivated cis/trans-isomerization.^65^ In vinyl halides, in particular, the halogen substituent (F, Cl, Br, I) introduces conical intersections between the $\pi \pi^{*}$ state and $n\pi^{*}$/ $n\sigma^{*}$ states, leading to efficient ultrafast photodissociation of the halogen–carbon bond.^66^ We employ attosecond transient absorption spectroscopy (ATAS) to simultaneously follow neutral and cationic states dynamics of the molecule after the interaction with the strong NIR field by probing the Br M edge. The experiment leverages a previous investigation,^67^ performed at coarser tens-of-femtoseconds temporal resolution, highlighting the great amount of information and detail accessed by attosecond spectroscopies. That work assigned features based on conventional intuition known at the time; in hindsight from the new theoretical work here, numerous assignments of the features in the earlier experimental spectra are reassessed.

## II. METHODS

**FIG. 1.**
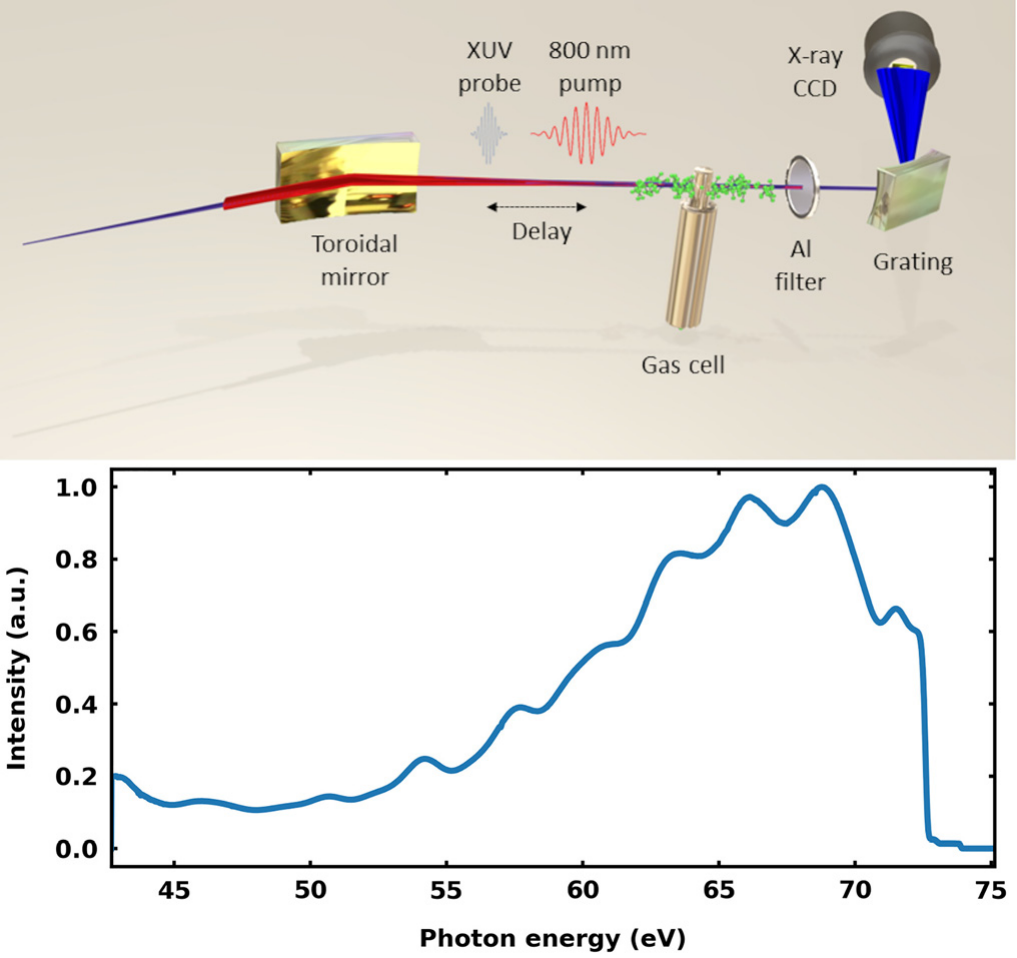
(Top) Schematic of the employed ATAS setup. After being delayed by a piezo-driven stage and collinearly recombined (not shown), the 800 nm fs pump and XUV attosecond (as) probe pulses are focused with a toroidal mirror into a static absorption gas cell, filled with vinyl bromide. The transmitted XUV light, after removal of the copropagating 800 nm radiation by an aluminum filter, is spectrally dispersed with a grating and imaged with a CCD camera. (Bottom) The XUV spectrum of the generated attosecond probe pulses. The sharp decrease at 72.7 eV is due to the employed aluminum filter.

The output of a carrier-envelope phase (CEP) stable Ti:sapphire system ( λ=800 nm), delivering 27 fs pulses with an energy of 2 mJ per pulse at 1 kHz, is spectrally broadened in a Ne-filled stretched hollow-core fiber. The pulses are then compressed using both a set of broadband chirped mirrors and a 1-mm-thick ammonium dihydrogen phosphide (ADP) crystal,^68^ yielding a compressed pulse duration of sub-4 fs. The pulses are then divided into a pump and probe arm using a 50:50 beam splitter. The employed ATAS pump-probe scheme is depicted in Fig. 1. The XUV-probe pulse is generated via HHG by focusing the few-cycle NIR pulses into a 3-mm-long gas cell backed with 30 Torr of Ar. Isolated attosecond pulses, covering the energy range between 55 and 73 eV, are obtained by the amplitude gating technique,^69^ as shown in Fig. 1. The residual NIR light is filtered out of the probe arm with a 200-nm-thick Al filter. According to previous streaking measurements,^68^ the XUV pulse duration is estimated to be 170 as. A toroidal mirror is used to focus the radiation into a static gas cell (3 mm interaction length) filled with 10 Torr of $C_{2}H_{3}\text{Br}$ (Sigma-Aldrich) at room temperature (298 K).

The NIR-pump pulse travels through a piezoelectric delay stage and is collinearly recombined with the XUV-probe with a hole mirror placed before the focusing toroidal mirror. On target, the intensity of the NIR-pump pulse, $I=2\times 10^{14}\;W\;\;{\text{cm}}^{-2}$, is high enough to trigger various competing processes, both in the neutral molecule (via frustrated tunnel ionization and multiphoton excitation) and in its cation (via strong-field ionization). The NIR-pump pulse is finally removed from the beam path after the interaction gas cell using a second 200-nm-thick Al filter. The XUV radiation transmitted through the interaction gas cell is analyzed with a home-built spectrograph, consisting of a dispersive, gold-coated, aberration-corrected concave XUV grating (Hitachi) and a back-illuminated x-ray-sensitive cooled CCD camera (Princeton Instruments). The energy resolving power of the spectrograph is estimated to be E/dE=1000 from previous experiments,^11^ yielding a resolution of 70 meV at 70 eV photon energy. A beam shutter is programmed to block the NIR pump arm in order to reference the $C_{2}H_{3}\text{Br}$ XUV absorption spectrum with and without the NIR pump pulse and measure the differential optical density, defined as the natural logarithm of the ratio between the spectra when the pump is on and off.

## III. COMPUTATIONAL DETAILS

### A. *Ab initio* level of theory

**FIG. 2.**
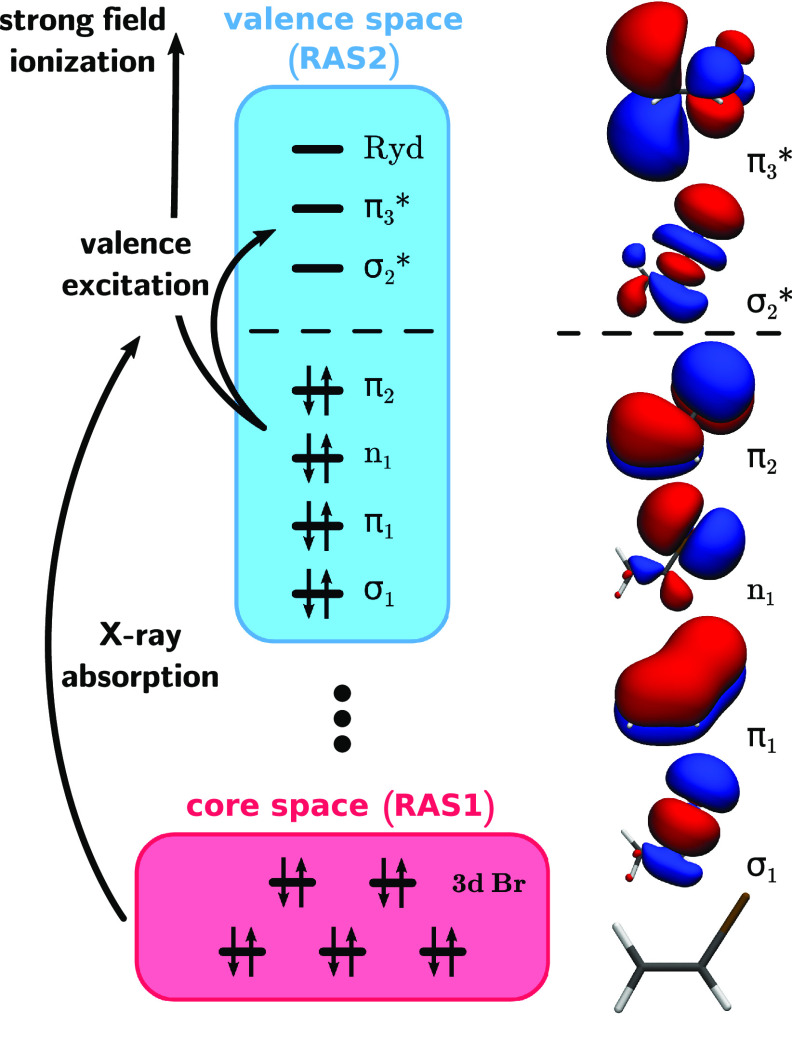
Diagram of the relevant excitations for an x-ray/XUV absorption spectrum. The RAS2 depicted in blue allows us to describe the valence excited states as well as cationic states after the interaction with the strong field. The RAS1 includes all five 3*d* bromine core orbitals and is shown in red. The combination of both RASs allows us to treat the x-ray/XUV absorption and simulate the XAS. The orbitals are shown with an isovalue of 0.02 and oriented according to the geometry at the bottom.

The electronic states of vinyl bromide were computed using the state-average complete active space self-consistent field method^70,71^ including five states in the state-averaging procedure (SA5-CASSCF). All calculations were carried out with the OpenMolcas^72,73^ program package using a modified ATZP basis set (m-ATZP). For more details about the modification and the validation of its use, see the supplementary material Sec. II B.^108^ An active space (AS) including eight electrons in seven orbitals [AS(8,7)] was employed. It includes the carbon–carbon double bond (*π*_1_, $\pi_{3}^{*}$) (Fig. 2), the carbon–bromine single bond (*σ*_1_, $\sigma_{2}^{*}$) as well as both remaining bromine 4*p* orbitals. One nonbonding orbital is part of a second *π* orbital (*π*_2_); the other one forms the lone-pair *n*_1_. To stabilize the active space, we also had to include one additional virtual orbital ( $\pi_{4}^{*}$), which has significant Rydberg (Ryd.) contributions. The complete AS is shown in Fig. S7 in the Supporting Information, and the main parts of it, except for the $\pi_{4}^{*}$/Ryd. orbital, are also in Fig. 2. An overview of the excited states of both neutral and cationic vinyl bromide, their vertical excitation energies, and electronic character is given in Table I. The states are ordered with increasing energy and are labeled according to their adiabatic state number at the Frank–Condon (FC) point.

**TABLE I.** Excited state electronic character and vertical excitation energies ΔE of neutral and cationic vinyl bromide. The excitation energy is given in units of eV relative to the neutral ground state (*S*_0_) at the Frank–Condon point. For the character of the cationic vinyl bromide, the orbitals that are partially occupied are listed.

Neutral			Cationic		
State	Character	ΔE (eV)	State	Character	ΔE (eV)
*T*_1_	$\pi_{2}\to \pi_{3}^{*}$	4.08	*D*_1_	*π*_2_	8.81
*T*_2_	$\pi_{2}\to \sigma_{2}^{*}$	6.26	*D*_2_	*n*_1_	9.72
*T*_3_	$n_{1}\to \sigma_{2}^{*}$	6.61	*D*_3_	*π*_1_	11.28
*S*_1_	$\pi_{2}\to \sigma_{2}^{*}$	6.67	*D*_4_	*σ*_1_	12.94
*S*_2_	$\pi_{2}\to \pi_{3}^{*}$	7.34	*D*_5_	$n_{1},\pi_{2},\pi_{3}^{*}$	14.26
*S*_3_	$n_{1}\to \sigma_{2}^{*}$	7.50			
*T*_4_	$n_{1}\to \pi_{3}^{*}$	7.53			
*S*_4_	$n_{1}\to \pi_{3}^{*}$	7.77			
*T*_5_	$\pi_{2}\to \pi_{4}^{*}$	8.16			

### B. Dynamics simulation

The NAMD of vinyl bromide were simulated using the surface hopping including arbitrary couplings (SHARC) program package.^74–76^ A set of 400 initial conditions (geometries and velocities) were generated based on a Wigner distribution^77,78^ at 0 K computed from harmonic vibrational frequencies in the optimized ground state minimum. The underlying optimization and frequency calculations were performed at the closed-shell coupled-cluster level of theory, including singles, doubles, and perturbative contributions of triples using the aug-cc-pVTZ basis set^79–83^ [CCSD(T)/aug-cc-pVTZ]. For the neutral species, a subset of 300 trajectories was randomly chosen and propagated starting in the bright $\pi \pi^{*}$ state (at the FC point *S*_2_). To relate to the excitation process, the initialization for the neutral species was done in the diagonal representation, where the spin-mixed, fully adiabatic states were obtained by diagonalizing the electronic Hamiltonian matrix, which itself is built up from five singlet and triplet states for the neutral species. In addition, all relevant spin–orbit interactions are included in the Hamiltonian matrix. To ensure excitation only to the bright $\pi \pi^{*}$ state, the transition dipole moment for each initial geometry is calculated as the selection criterion. For the cation, the trajectories were set up in the molecular Coulomb Hamiltonian (MCH) representation, where the states are represented in the basis of the eigenfunctions of the molecular Coulomb Hamiltonian, consisting of five doublet and quartet states. Sets of 50 randomly chosen trajectories were started each in the *D*_1_, *D*_2_, and *D*_3_ ionic states. In both cases, the propagation itself was performed in the diagonal representation. The necessary energies, gradients, spin–orbit and non-adiabatic couplings (SOC, NAC) were calculated on-the-fly at the SA5-CASSCF level of theory using the Molcas/OpenMolcas interface included in SHARC. The non-adiabatic transitions were treated within Tully's fewest switch trajectory surface hopping (TSH) algorithm^84^ as implemented in SHARC.^76^ The integration of the nuclear motion is done using the Velocity-Verlet algorithm with a maximal simulation time of 100 fs using a time step of 0.5 fs. The subsequent analysis of the trajectories of both species is done in the MCH representation.

### C. Simulation of x ray/XUV absorption spectra

The following describes, first, the general procedure how we calculated the static x-ray/XUV absorption spectrum (further abbreviated as only XAS) based on the RASPT2 ansatz and second, how we used the NAMD simulation to generate the transient XAS of vinyl bromide.

The algorithm for the calculation of the XAS is mainly based on the works of Josefsson and co-workers^26^ and Wang, Odelius, and Prendergast.^42^ Here, both the valence space of vinyl bromide and the excitation processes of the core electrons are described in the same theoretical framework of the restricted active space self-consistent field (RASSCF) method.^85,86^ RASSCF is an extension to the general CASSCF approach, where the active space is further partitioned into three subspaces RAS1, RAS2, and RAS3, with additional constraints applied to their occupation. In general, they can be systematically labeled RAS( n,l,m;i,j,k), where, *i*, *j*, and *k* are the number of orbitals in the RAS1, RAS2, and RAS3 subspaces, respectively, *n* is the total number of electrons in the active spaces, *l* the maximum number of holes allowed in the RAS1, and *m* the maximum number of electrons allowed in RAS3. For the RAS2, all possible configurations are allowed, making it analogous to the AS within CASSCF. For vinyl bromide, the RAS2 was built up similarly to the AS utilized in the NAMD. It also included the carbon–carbon double bond (*π*_1_, $\pi_{3}^{*}$), the carbon–bromine single bond (*σ*_1_, $\sigma_{2}^{*}$) as well as both remaining bromine 4*p* orbitals (*π*_2_, *n*_1_) and one additional virtual orbital with significant Rydberg contributions. For the RAS1, we included all five 3*d* orbitals of bromine and allowed for one hole. To enhance convergence within the RASSCF and to suppress unwanted orbital rotation with the valence space, the core orbitals were kept frozen to their shape from the Hartree–Fock calculation. The RAS3 was not utilized, thus resulting in RAS( 18,1,0;5,7,0) for neutral and RAS( 17,1,0;5,7,0) for cationic vinyl bromide. The diagram in Fig. 2 summarizes all relevant excitations and orbitals involved for the computational setup. As bromine is already subject to spin–orbit effects,^87^ relativistic effects were included and treated in two steps, both based on the Douglas–Kroll Hamiltonian.^88,89^ In the first step, within each spin symmetry the states were optimized in a state-averaged RASSCF procedure carried out with the relativistic atomic natural orbital basis (ANO-RCC),^90–94^ contracted to ATZP quality (ANO-RCC-ATZP). This is followed by a perturbative inclusion (PT2) of the dynamic electron correlation within the multistate RASPT2 method,^95–99^ where the default ionization-potential electron-affinity (IPEA) shift^100^ of 0.25 Hartree was used. An additional imaginary shift^101^ of 0.2 Hartree was applied to reduce problems with intruder states. In the second step, these “spin-free” states were used as a basis in the restricted active space state interaction (RASSI) method,^102–104^ where the spin–orbit coupling (SOC) was treated with the use of the atomic mean-field spin–orbit integrals (AMFI).^105^

For the simulation of the transient XAS discussed further below, we utilized the geometric information from the on-the-fly trajectories. For every 3 fs snapshot, the XAS spectrum is calculated. Note that the multi-configurational wave function from the dynamics simulation could not be reused as it did not include all orbitals necessary for the proper description of the XAS spectrum. The spectrum requires a new active space including the five bromine 3*d* core-orbitals and the extended ANO-RCC-ATZP basis set.

For each single XAS calculation, seven singlet and eight triplet valence excited states for the neutral and five-doublet and three-quartet valence excited states for the cationic species are calculated. Additional 40 core excited states for both species, and all four multiplicities, were calculated by enforcing one hole in RAS1. This results in a total of 95 states for the neutral and 88 states for the cationic species. The interaction between all these states, including SOC, was treated within in the RASSI method. Note that since the valence and core excited states are obtained from different calculations, their corresponding wave functions are not inevitably orthogonal. This was already mentioned by Wang, Odelius, and Prendergast.^42^ Following their proposed procedure, we applied the original correction to the overall Hamiltonian as introduced in Ref. 104. The next step is to match the valence states from the XAS calculation with the valence states from the trajectory calculations to obtain the transient XAS spectrum for the active state. In the case of the neutral vinyl bromide, we used the dipole moment vector of the active state of the trajectory for the matching. For the cationic case, we found it to be sufficient to use the state number (index) of the active state. The oscillator strengths that are related to the matched valence excited state were then used to simulate the transient XAS. The vertical transition energies were obtained from the spin–orbit RASSI states (SO-RASSI) and no energy shift was applied. An overall Gaussian convolution with σ=0.1 eV was applied to broaden the calculated spectrum. For each time step, the convoluted spectra were averaged over all geometries and then normalized to the maximum of all calculated time steps. Furthermore, the XAS of the *S*_0_ ground state at time t=0 fs was subtracted to simulate the ground state bleach in the energy range upwards of 70.0 eV. A flow chart of the complete procedure as well as example Molcas inputs for the necessary calculations can be found in the supplementary material Sec. II G.^108^

## IV. RESULTS AND DISCUSSION

### A. Spectroscopic assignments of the transient absorption spectra

**FIG. 3.**
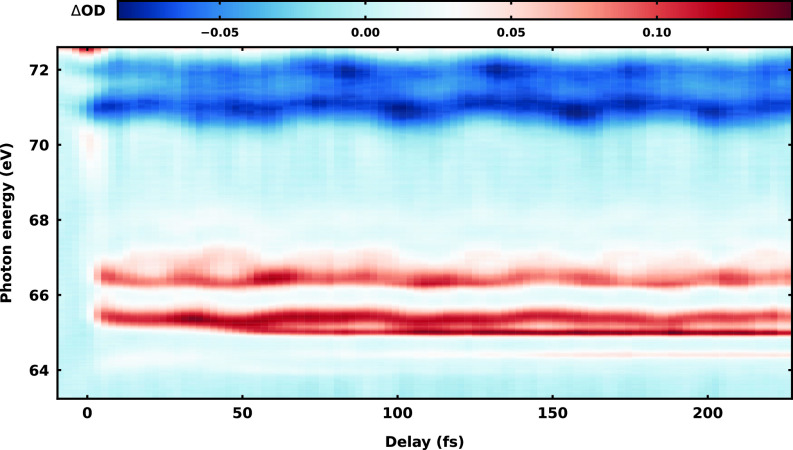
XUV transient absorption spectrogram of $C_{2}H_{3}\text{Br}$ exposed to strong-field interaction with a sub-4 fs NIR pulse centered at λ=800 nm, with a peak intensity of $I=2\times 10^{14}\;W\;\;{\text{cm}}^{-2}$. The transient is recorded by collecting the differential optical density at different pump-probe delays, with a step size of 2.3 fs. For negative delays, the XUV pulse arrives first, and for positive delays the NIR pulse arrives first.

The transient absorption spectrogram, recorded by collecting the differential optical density at different pump-probe delays with 2.3 fs step size, is shown in Fig. 3. Further details on data processing are provided in Sec. I A of the supplementary material.^108^ The spectral range of the attosecond XUV-probe pulse covers the pre-edge region of the M_4,5_ edge of Br (located at 75.3 eV), corresponding to the excitation of a Br 3*d* electron. The applied NIR pump pulse launches multiple strong-field-initiated dynamics in vinyl bromide. The main process triggered is strong-field ionization, but neutral valence excited-state vinyl bromide can also be formed by a multiphoton process. As shown by closely related studies on methyl iodide,^12^ methyl bromide,^13^ and vinyl bromide,^67^ both processes can occur under these conditions, leading to a rich transient absorption spectrum.

The bleaching signals at 71.0 and 71.9 eV are due to the depletion of the ground state of the molecule after the interaction with the strong NIR field. They appear as a doublet, as is the case for features discussed below, due to the 0.9 eV spin–orbit splitting of $3d_{5/2}$ and $3d_{3/2}$ states in Br.^106^ The main doublet appearing at 65.5 and 66.4 eV as well as the weaker signals up to 70 eV has been assigned to ionic states including also the dication.^67^ Following the previous assignment, the main doublet corresponds to the excitation of the 3*d* Br electron of the ionic vinyl bromide in the *D*_2_ state (for details of the nomenclature see Table I). At long temporal delays ( t>100 fs), sharp atomic Br lines at 64.4 and 65.0 eV indicate an ultrafast dissociation of the molecule or ion.

Pronounced, periodic, delay-dependent spectral modulations of the absorption are visible both in the ground state bleaching and cationic state signals. In both cases, they reflect vibrational wavepacket motion, as already pointed out in a number of closely related investigations.^12,13^ The delay-dependent spectral first-moment of the absorption signal in the range 70.3–71.5 eV reveals a clear oscillation (see Sec. I B of the supplementary material^108^) with a period of 52.5 fs. The corresponding frequency of 635 ${\text{cm}}^{-1}$ agrees well with the C–Br eigenmode *ν*_3_ in the ground electronic state of neutral $C_{2}H_{3}\text{Br}$ (see Table S7 in the supplementary material^108^ and Ref. 107). Given the initial phase of $\phi_{0}=-\pi$ (cosine-like wave), the wavepacket is likely to be launched by a bond softening mechanism, as detailed by Wei *et al.*^12^ This fine mapping in the energy domain of the vibrational wavepacket motion is one of the unique aspects of ATAS.

A more prominent example of this resolving capability is provided by the dynamics of the cationic *D*_2_ state. The spectral first-moment of the signal in the range 65.2–65.7 eV is in this case composed of the sum of two dephased sinusoidals (see Sec. I B of the supplementary material^108^) with frequencies of 480 and 1220 ${\text{cm}}^{-1}$, corresponding to the *ν*_3_ (C–Br, stretching) and *ν*_7_ (CCH, bending) eigenmodes, respectively (see Table S7 in the supplementary material^108^). The activation of the *ν*_3_ and *ν*_7_ modes in the ionization process to the *D*_2_ state of cationic vinyl bromide has already been observed and understood in terms of the displacive nature of the vertical Franck–Condon excitation.^107^ Indeed, the equilibrium geometry of the *D*_2_ cationic state features two main differences compared to the neutral ground state: an increase in C–Br bond length (by 0.06 Å) and in CCH angle (by 9.10%) (see Table S9 in the supplementary material^108^). In comparison with a spectral domain spectroscopy method, here, ATAS allows one to completely map in space and time this multidimensional motion, giving access to the phase of the different contributions, relative to the instant and to each other.

**FIG. 4.**
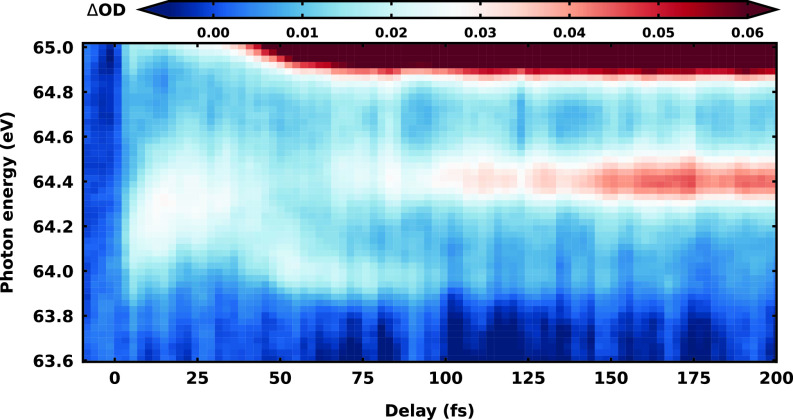
Zoom of the transient absorption spectrogram showing the new feature in the energy range between 64.0 and 65.0 eV.

In the energy region of 64.0 and 65.0 eV, a previously not reported short-lived ( t<100 fs) feature centered at 64.3 eV at t=0 fs is observed. A zoom of the transient absorption spectrogram showing this new feature can be found in Fig. 4. It is immediately evident that, compared to the oscillatory nature of the vibrational coherences explained above, the temporal behavior of the feature is more complicated. After an initial energy upshift of the absorption feature, from 64.30 to 64.45 eV within the first 25 fs, the signal bifurcates around t=30 fs. The main branch downshifts in energy by 0.35 eV in the following 10 fs, reaching the asymptotic value of the atomic Br M_4,5_ transition^106^ $P_{1/2}\to D_{5/2}$ at 64.1 eV at t=40 fs. It is important to note that the 1/2 to 5/2 transition is strictly dipole forbidden in the atomic limit due to spin–orbit selection rules, but, in the molecule, because of spin–orbit coupling among electrons, the restriction is removed. In fact, the signal at 64.1 eV vanishes within the following 40 fs, in concomitance with the birth and rise to asymptotic intensity of the allowed atomic Br transitions $P_{3/2}\to D_{5/2}$ (at 64.4 eV) and $P_{1/2}\to D_{3/2}$ (at 65.0 eV). The transient behavior, combined with the delayed rise of the atomic Br dissociation products, clearly indicates that we are observing an ultrafast dissociation process. The observation appears in close connection to the recently investigated ATAS of methyl bromide.^13^ There, the feature was assigned to a neutral excited state populated by multiphoton process and the bifurcation was attributed to the non-adiabatic passage through a conical intersection. In the case of vinyl bromide, it is not straightforward to distinguish in the spectroscopic data whether this signal originates from an ionic or neutral dissociation process. In the critical range between 64.1 and 65.0 eV, the previous investigation^67^ of vinyl bromide predicted the appearance of a signal due to the second-excited cationic state (*D*_3_) according to Koopman's theorem, which could, however, not be resolved at that time. Applying the same simple Koopman's picture on neutral vinyl bromide, a signal in the same range is expected, which could be assigned to an electronically excited $\pi \pi^{*}$ state of the neutral.

In fact, the first UV absorption band of the molecule is composed of a very broad ( >0.5 eV) feature centered around 6.5 eV;^107^ this is in range with the distance between the signal under consideration and the ground state bleach doublet. In the present strong-field pump scenario, such an excited state could be reached in principle via a four-photon-process. Noteworthy, an ultrafast C–Br bond-rupture channel of the neutral has been inferred from 193 nm photodissociation studies.^65^ In order to disentangle between the two possibilities, neutral vs ion as well as to explain the complete transient absorption spectrogram, we simulated the transient XAS spectra for neutral and ionic vinyl bromide.

### B. Excited state dynamics of the neutral vinyl bromide

In order to initiate the neutral dynamics, we approximate the multiphoton process as a one-photon excitation. We start 300 trajectories in the bright excited state (for more details see Sec. III B). Using the default selection criteria provided by SHARC, 189 trajectories were taken into account for the analysis of the excited state dynamics and calculation of the XAS spectrum. The initial and final populated adiabatic states are summarized in Table II.

**TABLE II.** Population of the ten calculated neutral states at the start and end point of the simulation. All percentages are given with respect to the total number of analyzed trajectories.

State	Start	End	State	Start	End
*S*_0_	0%	8%	*T*_1_	0%	18%
*S*_1_	4%	8%	*T*_2_	0%	18%
*S*_2_	38%	5%	*T*_3_	2%	16%
*S*_3_	34%	2%	*T*_4_	16%	11%
*S*_4_	3%	2%	*T*_5_	3%	12%

The excitation to the bright $\pi \pi^{*}$ state corresponds to an initial population of 38% in the *S*_2_ state and 34% in the *S*_3_ state. Since Br is a heavy atom, where spin–orbit effects arise, we also see a slightly smaller initial population of 16% in the *T*_3_ state. After excitation, all trajectories show non-adiabatic as well as spin–orbit transitions within the simulation time of 100 fs. The overall change in population for all states is shown in Fig. S9 in the supplementary material.^108^ In the first 20 fs, population is mainly transferred from the three initial states into the triplet states *T*_2_ to *T*_5_. After this initial period of 20 fs, also, the population of the *S*_0_ and *T*_1_ states increases, where *T*_1_ is populated faster. Over the next 80 fs, the general trend of population transfer to the triplet states stays the same. Only the population of the *S*_0_ state increases to about 8%. At the end of the simulation, most of the population (about 75%) can be found in one of the triplet states. The remaining 25% is distributed over all five singlet states. In each multiplicity, the first two states (*S*_0_, *S*_1_, *T*_1_, *T*_2_) are populated the most.

**FIG. 5.**
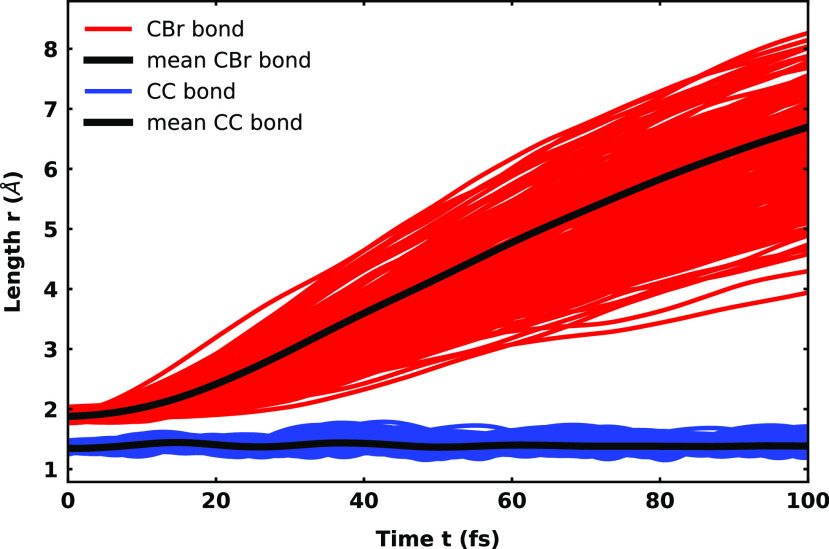
Temporal evolution of the CC double bond and the CBr bond for the 189 analyzed trajectories of neutral vinyl bromide.

No matter in what electronic state the trajectories end up, their final geometries show a quite uniform picture. For elucidation, the temporal evolution of both the C=C double bond and the C–Br bond is shown in Fig. 5. All analyzed trajectories are of dissociative nature, showing on average a doubling of the initial C–Br bond length after about 50 fs. No oscillation in the C–Br bond and only a very weak one in the C=C double bond are observed. The fragments reveal that only homolytic dissociation occurs. Two types of electronic configuration are present, which differ only in the position of the unpaired electron in the vinyl radical. In the case of the $S_{0-2}$ and $T_{1-3}$ states, the unpaired electron resides in the 2*p* orbital of carbon that was part of the former *σ* C–Br bond ( $H_{2}C=C^{•}H$). For states $S_{3,4}$ and $T_{4,5}$, the electron is in the *π*_2_ orbital of the C=C bond, with the carbon 2*p* orbital now being doubly occupied ( $H_{2}C\overset{•}{–}\text{CH}$). For both channels, the electron on the Br radical sits in one of the three now degenerate 3*p* orbitals.

**FIG. 6.**
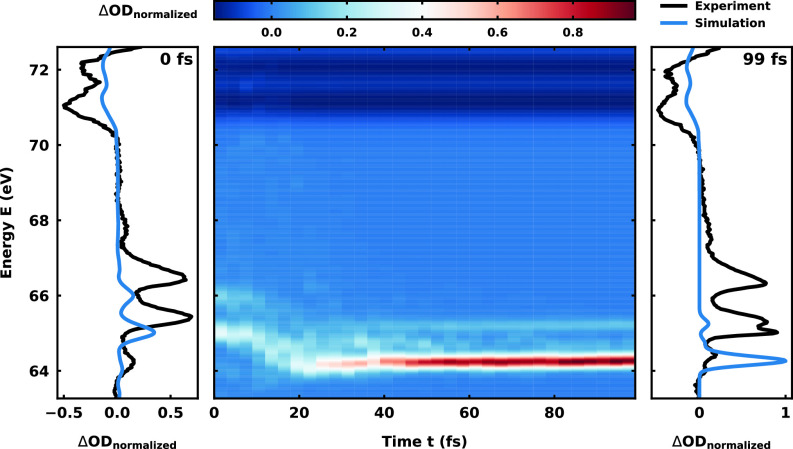
Simulated transient XAS of the neutral trajectories starting in the bright $\pi \pi^{*}$ state. On the left and right, the spectrum for time *t* = 0 and t=99 fs, respectively, is shown in blue overlaid with the experimental transient absorption spectogram in black at delays *d* = 6.8 and d=107.6 fs.

Combining the data from the analyzed trajectories with our RASPT2 ansatz, we simulated the transient XAS of neutral vinyl bromide. The resulting time-resolved spectrum is plotted in Fig. 6. First one has to note that the simulated spectrum matches the experimental energy range quite well, keeping in mind that we did not apply a shift to the excitation energies. The energetic position of the ground state bleach at 71.0–72.5 eV matches very well. The temporal modulation is missing, since we simply subtracted the XAS of the *S*_0_ ground state at time t=0 fs as mentioned in Sec. III C. In the range of 64.0–65.0 eV, prominent features occur that are predicted by Koopman's theorem. All of them are shifting approximately 1 eV toward lower energies within the first 20 fs. Thereafter, two sharp features, a stronger one at 64.3 eV and a significantly weaker one at 65 eV, appear. After another 10–20 fs, all signals remain constant in energy and the feature at low energy becomes more and more pronounced. Since nearly all trajectories are dissociated at this point in time, these two remaining constant signals are the spin-allowed transitions of atomic bromine. From both transitions, the lower lying $\text{Br}(P_{3/2})\to \text{Br}(D_{5/2})$ is more dominant. Comparison to the experimental spectrum reveals that the signals from the neutral species alone cannot explain the experimentally observed pattern in this energy range. Thus, signal contributions from the ionic species are required in this region as well as evidently also in the higher energy part above 65.5 eV.

### C. Dynamics of the cationic vinyl bromide

Next, we examined the excited state dynamics of cationic vinyl bromide. As discussed in the previous work,^67^ the first three ionic states (*D*_1_, *D*_2_, and *D*_3_) are accessible with the pump pulse used. Accordingly, we started 150 trajectories equally distributed in each of these states. For the analysis, 82 out of the 150 calculated trajectories were taken into account, using the same selection criteria as for the neutral case. The 82 trajectories break down to 11 that started in the *D*_1_ state, 26 in the *D*_2_ state, and 45 in the *D*_3_ state. The overall change in population for all 82 trajectories is shown in Fig. S17 in the supplementary material.^108^ All three states are bound states, with, however, decreasing dissociation energies, reflecting the increasing weakening of the C=C double bond with ionization energy. Contrary to the neutral case, their dynamics differ depending on the initial state and will be discussed individually in the following.

#### 1. Starting from the D_1_ state

The electronic character of the *D*_1_ state is characterized by an ionization from the *π*_2_ orbital (see Figs. 2 and S7). The complete population remains in the *D*_1_ state for the entire simulation time. There is no interaction with either the other doublet or quartet states (see Fig. S11). None of the analyzed trajectories dissociate. This is in good agreement with the result of a relaxed scan along the C–Br bond that predicts a barrier of about 1.5 eV for the dissociation (see Fig. S28 and the computational details Sec. V B in the supplementary material1^108^). We observe an oscillation of the C=C bond with a period of about T=25 fs and of the C–Br bond with a period of T=50 fs (see Fig. S12). The launching of both oscillations is reasonable, since the affected *π*_2_ orbital has contributions from both the C=C double bond as well as one of the bromine 4*p* orbitals.

#### 2. Starting from the D_2_ state

For the *D*_2_ state, the electron hole is generated in the *n*_1_ lone-pair orbital of the bromine. Again, most of the population remains in the initial state *D*_2_, with only a small part, less than 5%, being transferred into the *D*_1_ state. As for the *D*_1_ state, there is no further interaction with the other electronic states (see Fig. S13) and none of the analyzed trajectories dissociate. The barrier toward direct dissociation is about 1 eV (see relaxed scan Fig. S28). Since ionization from orbital *n*_1_ mainly affects the C–Br bond, a pronounced oscillation along this bond with a period of about T=75 fs (see Fig. S14) is observed. The bond elongates from the initial 1.9 to roughly 2.08 Å. Only a weak oscillation, with a period of about T=20 fs, that dampens over the simulation time, is present in the C=C bond. This bond is hardly affected and its bond length shortens only marginally to about 1.31 Å. In summary, the first two ionic states exclusively show bound state vibrational dynamics.

#### 3. Starting from the D_3_ state

**FIG. 7.**
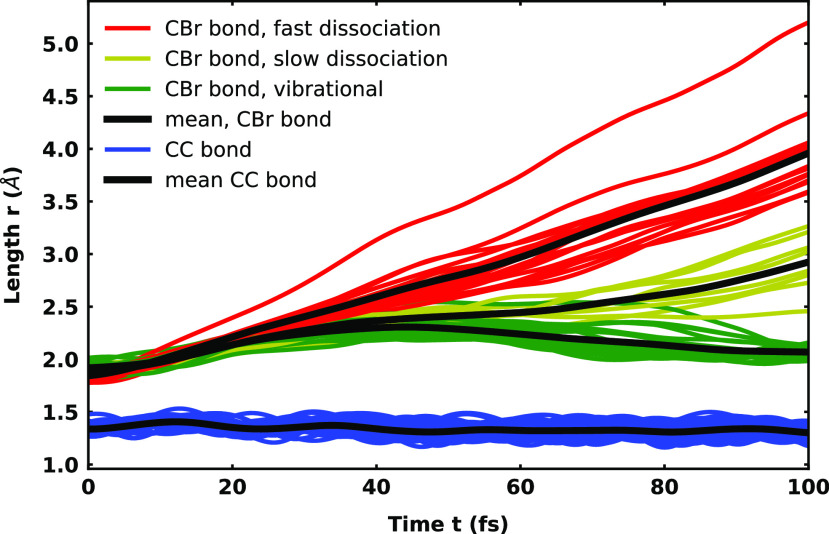
Temporal evolution of the C=C double bond and the C–Br bond for the 45 analyzed trajectories that started in the *D*_3_ state of the cationic vinyl bromide. For the C–Br bond, the corresponding trajectories are color coded according to their observed behavior. Only vibrational trajectories are shown in green, slow dissociating ones in yellow, and (fast) dissociating trajectories in red. The average of the C–Br bond length was only calculated for the subset of the trajectories.

For the *D*_3_ state, the hole is created in the C=C bonding *π*_1_ orbital (see Figs. 2 and S7), which has contributions from the C=C double bond and the bromine 4*p* orbital. The C=C double bond as well as the C–Br bond are now strongly weakened. Accordingly, the relaxed scan (see Fig. S28) predicts a small barrier of only 0.2 eV for a direct dissociation. The temporal evolution of the *D*_3_ population (see Fig. S15) shows a slow but steady decay due to population transfer to the *D*_2_ and *D*_1_ state after the first 20 fs. There is also a slight intermediate interaction with the *D*_4_ state between 20 and 60 fs, but again, there is no further significant interaction with the remaining electronic states. After 100 fs, more than 80% of the population is still in the *D*_3_ and about 10% in each of the *D*_2_ and *D*_1_ states. In the barrier region, transitions to the *D*_4_ state via conical intersections are possible (see Sec. V of the supplementary material^108^ for the optimized structure). After the barrier, the *D*_3_ and *D*_4_ state switch character. For the new character, the *σ*_1_ orbital of the C–Br bond is only singly occupied leading to a repulsive potential correlating with the dissociation channel of the *D*_1_ or *D*_2_ state. Judging from the small barrier compared to both other cationic states, one would expect most of the trajectories to dissociate. And indeed, the dynamics starting in the *D*_3_ state reveal a more complex situation regarding the temporal evolution of the C–Br bond (see Fig. 7). Three types of dynamics were recognized, the pure vibrational (green), the slow dissociative (yellow), and the fast dissociative (red). The classification was done with regard to the length of the C–Br bond at the end of the simulation. Trajectories with a bond length longer than 3.5 Å were classified as fast. The range 3.5–2.2 Å was classified as slow and trajectories with shorter bond length as vibrational. The final distribution regarding these three types of dynamics is listed in Table III. About half of the analyzed trajectories dissociate within the simulation time of 100 fs, with the other half showing purely vibrational dynamics. For all trajectories, the C=C bond shows oscillation with a period of about T=25 fs. The purely vibrational trajectories indicate a slow oscillation with about T=100 fs in the C–Br bond. All of them stay in the *D*_3_ state, not being able to overcome the barrier. For the fast dissociating trajectories, at about 40 fs and a C–Br bond length of about 2.59 Å, we can see a clear separation from the rest of the trajectories. In the case of the slow dissociating trajectories, the separation happens later at 60 fs at a bond length of about 2.37 Å.

**TABLE III.** Observed dynamics of the 82 analyzed trajectories for cationic vinyl bromide. In the table heading, Dissociation is abbreviated as Diss.

Initial state	Vibrational	Slow Diss.	Fast Diss.
*D*_1_	11	0	0
*D*_2_	26	0	0
*D*_3_	21	8	16

In general, the dissociation in the *D*_3_ state is considerably slower compared to the neutral vinyl bromide, where we observed the onset of dissociation after 50 fs. For the cationic states, the bond cleaves heterolytically, leaving a neutral bromine radical and a vinyl cation. For the dissociation channel of all three states, *D*_1_, *D*_2_, or *D*_3_, the unpaired electron is located in one of the three now degenerate Br 3*p* orbitals.

**FIG. 8.**
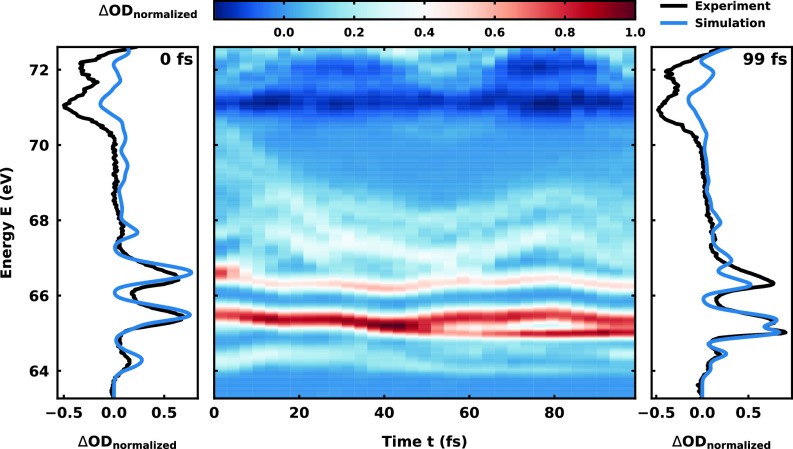
Simulated transient XAS of the cationic trajectories starting in the *D*_1_, *D*_2_, and *D*_3_ states. On the left and right, the spectrum for time *t* = 0 and t=99 fs, respectively, is shown in blue overlaid with the experimental transient absorption spectrogram in black at delays *d* = 6.8 and d=107.6 fs. Additional overlay plots at times 24, 48, and 72 fs as well as separated XAS of the different starting states and different observed dynamics are depicted in the supplementary material, Sec. IV.

Again using the described ansatz in Sec. III C and the data from the 82 analyzed trajectories, we simulated the complete transient XAS for the cationic states, which are depicted in Fig. 8. Comparing it to the experimental spectrum in Fig. 3, the overall resemblance in the measured energy range is strikingly good. To assign the different features of the spectrum, we additionally simulated the XAS only for a subset of the trajectories depending on their starting state, *D*_1_ (Fig. S21), *D*_2_ (Fig. S22), and *D*_3_ (Fig. S23). In the case of the *D*_3_, it is further separated according to the bound and dissociative dynamics (Figs. S24–S26).

In the following, we discuss the significant features of the spectrum in the context of the corresponding dynamical processes of vinyl bromide. Starting from the top, we observe the same ground state bleach as for the neutral states, but this time the intensity distribution is modulated by contributions from the *D*_1_ signal. The signals in the energy range 67–69 eV can be attributed to the *D*_1_ state. Although it is difficult to see the peaks in the complete spectrum, Fig. S21 clearly shows the pronounced doublet signal at 66.7 and 67.7 eV, the former with a higher, about twice, intensity compared to the latter. This doublet arises from the already mentioned spin–orbit splitting of the $3d_{5/2}$ and $3d_{3/2}$ orbitals of Br. Both signals oscillate with a period of about T=50 fs, which is in good agreement with the observed oscillation of the C–Br bond in the trajectories. In correspondence to the shortening of the C–Br bond during the oscillation, the signals shift to higher energies, reaching a maximum of about 67.7 and 68.7 eV in a half period. This shift can be explained by the electronic character of the *D*_1_ state. It has a hole in the *π*_2_ orbital, which has anti-bonding character with respect to the C–Br bond. Hence, shortening the bond destabilizes the *π*_2_ orbital and results in higher XUV excitation energies.

The next features at 65.5 and 66.5 eV in Fig. 8 are the most intense ones of the spectrum and can be attributed mainly to the *D*_2_ state by comparison to the *D*_2_ XAS (see Fig. S22). Here, the bromine-induced doublet structure shows most clearly and the energetically higher signal is less intense. The slight modulations with periods of about *T* = 80 and T=25 fs can be assigned to the C–Br and C=C bond vibrations in the *D*_2_ trajectories. With the elongation of the C–Br bond the doublet shifts toward lower energies. The elongation stabilizes the *n*_1_ lone-pair orbital and thus the *D*_2_ state, resulting in lower core excitation energies. This shift of about 0.3 eV is considerably smaller compared to the 1 eV of the *D*_1_ signal. Summarizing, the signals from both the *D*_1_ and *D*_2_ states show clear oscillations, but neither reproduce the characteristic splitting of the intense 65.5 eV peak at about 50 fs nor the weak band around 64 eV. These features must come from the *D*_3_ state.

The combined XAS for all *D*_3_ trajectories is shown in Fig. S23. The weak signals between 66 and 70 eV correspond to the core excitation into the $\sigma^{*}$ orbital of the C–Br bond. Again, the spectrum is dominated by the distinct doublet, now at 64.3 and 65.2 eV. In contrast to the previously discussed oscillating features, these signal traces become constant after about half an oscillation period at 40 fs and split up into four contributions at 64.0, 64.5, 65.0, and 65.5 eV with different intensities. They can be assigned to the various processes observed in the *D*_3_ trajectories.

The XAS for the purely vibrational bound state dynamics (see Fig. S24) shows weak signals between 66 and 70 eV and the prominent doublet peak at 64.3 and 65.2 eV, both oscillating with the period of the C–Br bond. The phase shift originates from the different final orbitals that are excited to. Bond elongation stabilizes the $\sigma^{*}$ orbital resulting in a down-shift of the weak signal. Simultaneously, the singly occupied *π*_1_ orbital is destabilized leading to an up-shift of the doublet signal.

For the XAS of the fast dissociating trajectories (see Fig. S26), the same doublet splitting at 64.3 and 65.2 eV occurs. After the initial first half of the oscillation period, the signal changes drastically. Both peaks become constant at an energy of 64.0 and 65.0 eV, and a strong increase in intensity for the energetically higher signal at 65.0 eV is observed while the other one is fading. This change in intensity and shape reflects the transition from the “molecular” bromine to the atomic bromine. With increasing distance between the bromine and the vinyl cation, the Br character becomes progressively more atom-like. From the two spin-allowed transitions $\text{Br}(P_{3/2})\to \text{Br}(D_{5/2})$ and $\text{Br}(P_{1/2})\to \text{Br}(D_{3/2})$, dominantly the higher lying transition $\text{Br}(P_{1/2})\to \text{Br}(D_{3/2})$ survives. Thus, in the cationic dissociation, spin–orbit excited $\text{Br}(P_{1/2})$ atoms are mostly generated, which is in excellent agreement with the experiment.

From the discussion of the XAS from the bound and the fast dissociating *D*_3_ trajectories, one would expect to see a mixture of their distinct signals in the XAS of the slow dissociating ones. Indeed, in the first 50 fs, Fig. S25 only shows the oscillating signals of the bound trajectories. In the time between 50 and 80 fs, the signals from the dissociating trajectories start to overlap converting into the constant signals of the atomic bromine at 64.0 and 65.0 eV.

In summary, the prominent features between 64 and 66 eV of the total XAS (Fig. 8) originate from overlapping signals of the cationic vinyl bromide. The signal is composed of spectral features of the non-dissociative dynamics in the *D*_2_ and *D*_3_ states and of the spin–orbit excited atomic Br generated in the dissociation of *D*_3_. This assignment relies on the fact that in vinyl bromide, the dynamics in the neutral states can be well distinguished from the dynamics in the cationic states, which can also be resolved in the XAS spectrum. In the accessible neutral state, a $\pi \pi^{*}$ excitation is immediately followed by a transition from the $\pi \pi^{*}$ to the $n\sigma^{*}$ configuration initiating barrierless dissociation. In contrast, for the cationic case, the hole is generated in different orbitals for the different states. The first two cationic states are bound due to being well separated in energy from dissociation channels. However, for *D*_3_ a slow transfer of the hole from the *π* into the *σ* orbital weakens the C–Br bond and leads to dissociation.

## V. CONCLUSION

We introduced a joint experimental and theoretical approach to follow in real-time the electronic structure change in molecules via attosecond transient absorption spectroscopy. It is then applied to study the ultrafast dynamics of vinyl bromide, after strong-field excitation from a few-cycle pulse centered at 800 nm. The remarkable agreement between the experimental and the calculated ATAS traces proves the high fidelity of the RASPT2 ansatz in combination with NAMD. Thus, it is possible to remove one of the two main bottlenecks historically ascribed to the employed methodology: the difficulty in data interpretation due to multiplet effects in probing inner valence rather than genuine core level states. Based on the NAMD trajectories, we calculated the state-specific XAS spectra and their temporal evolution. This allows for detailed insights into the electronic-state-resolved dynamics. In particular, we were able to clearly assign the experimentally observed spectrum to the dynamics of the first three electronic states of the cation, *D*_1_, *D*_2_, and *D*_3_. The first two states remain bound, allowing us to time-resolve the ensuing multidimensional vibrational dynamics with high sensitivity. The second excited cationic state *D*_3_, instead, presents richer dynamics. In addition to the pure vibrational motion, fast and slow dissociation channels also appear, leading to ultrafast rupture of the halogen–carbon bond in 50% of the calculated trajectories. No significant ultrafast relaxation channel from *D*_2_ to *D*_1_ has been observed, which is different from the very recent results on strong-field ionized ethylene.^22^ Each of the above-mentioned channels has a clear fingerprint in the ATAS spectrum, and they are unambiguously assigned thanks to the theoretical comparison. Extensions of the described methodology are imaginable both on the experimental and theoretical side. The combination of attosecond absorption and charged-particle-based spectroscopies (angle-integrated, via time-of-flight, or even angle-resolved, via velocity-map-imaging spectroscopy) would allow for an even greater insight into the different relaxation and fragmentation channels, providing a multidimensional space of correlated observables. In order to circumvent the issue of the non-resonant and non-perturbative nature, one could explicitly simulate the interaction of the molecule with the strong NIR field. A suitable approach would be the recently developed R-matrix ansatz^23,24^ or the B-Spline Restricted Correlation Space - Algebraic Diagrammatic Construction approach.^25^ Overall, the platform here presented can be readily applied to single-photon-excitation studies and to the soft x-ray spectral region, where the presence of carbon, nitrogen, and oxygen edges allows for molecular ultrafast studies with unprecedented time resolution.

## AUTHORS' CONTRIBUTIONS

F.R. and M.R. contributed equally to this work.

## Data Availability

The data that support the findings of this study are available from the corresponding author upon reasonable request.
